# Role of foreign direct investments in agriculture, forestry and fishing in developing countries

**DOI:** 10.1186/s43093-022-00164-2

**Published:** 2022-10-20

**Authors:** Linus Nyiwul, Niraj P. Koirala

**Affiliations:** 1 Department of Economics, Economics & Africana Studies, Gettysburg College, 300 North Washington St., Gettysburg, PA 17325 USA; 2 Department of Economics and Statistics, California State University Los Angeles, Los Angeles, USA

**Keywords:** FDI, Agriculture, Panel VAR, Food security, Developing countries, C32, F21, F35, O11, Q14

## Abstract

The primary sector is vital for growth and sustainable development in emerging countries. The combined effects of COVID-19 and geopolitical uncertainty on capital flows are likely to have profound impacts on many developing countries. In particular, decreased capital inflows into agriculture will negatively affect food security and growth. However, there remain limited literature on the role of capital inflows in this sector. In this paper, we examine the role that foreign capital inflows play in the development of the agricultural, forestry and fishing sectors in developing countries. Specifically, we use the panel vector autoregression approach that accounts for endogeneity. Using data from sixteen developing economies, we find that there exists bidirectional causality between foreign direct investments in agriculture, forestry and fishing and value added in these sectors. These bidirectional relationships reflect a cyclical effect between FDI and value added in the agriculture, forestry and fishing. The effect of FDI on value added in agriculture, forestry and fishing remains positive for up to five years in our model. This implies FDI has a medium- to long-term positive impact on value added in agriculture, forestry and fishing. The implication of this result is that countries with currently high FDI transaction costs or that have a generally less conducive investment environment can improve agriculture by eliminating these obstacles. This is because FDIs can lead to improved technologies and technical expertise, practices, management and other systems that benefit the host countries.

## Introduction

Food security and environmental quality are crucial requirements for sustainable development. Investments that address both can enhance growth opportunities in poor and developing economies, where the contribution of agriculture to the economy tends to be relatively large and sustains a disproportionately large share of the population. Castañeda et al. [10] estimate that 65% of poor-working adults earn a living via agriculture. The share of agriculture to global gross domestic product (GDP) is estimated to be 4% but compared to other sectors, the growth in this sector can be two to four times more effective in raising incomes among the poorest [50] and two to three times more effective in reducing poverty in Africa [51].

The 2021 Food and Agricultural Organization (FAO) statistical yearbook estimates the global value added generated by agriculture, forestry and fishing to be US$3.5 trillion in 2018, growing by 73% in real terms between 2000 and 2019 [17]. In Africa, the value added more than doubled in the same period and Asia accounted for sixty-four percent world total value added benefit in global agriculture, forestry and fishing in 2019. In terms of employment, the share of the global workforce in agriculture, forestry and fishing was twenty-seven percent in 2020, representing 874 million people. In Africa, agricultural employment increased to 224 million people, around 200 million people in China and the same number in India work in agriculture [17]. At the same time, climate change poses significant risks to this sector. Smith et al. [48], under the auspices of the IPCC, estimate that agriculture, forestry and other land use are the source of around twenty-five percent of anthropogenic emissions of carbon dioxide (CO_2_), methane (CH_4_) and nitrous oxide (N_2_O). Mitigating and adapting to climate change, improving food security, enhancing and maximizing employment, value added and growth will require substantial investments in the sector. The demand for food is estimated to increase by seventy percent by 2050 [14]. According to the World Bank [56], this growth in food demand will require at least $80 billion annual investments, and most of this will need to be sourced from the private sector. However, financial sector institutions in developing countries tend to lend a disproportionately lower share of their loan portfolios to agriculture compared to the agriculture sector’s share of GDP [56]. One way to alleviate this is to attract foreign direct investments into the sector. While the data indicate increasingly high levels of capital inflows into the sector in developing countries, there is surprisingly limited literature examining the contribution of these flows in the agricultural sector. The goal of this paper is to contribute to filling this gap in the literature. It is worth noting that the limited literature on this issue is in line with the broader lack of literature on effective interventions in the agricultural sector, as indicated by findings in Stewart et al. [47].

Specifically, we investigate the role of foreign direct investments in agriculture, forestry and fishing in developing countries between 2001 and 2020. Our goal is to identify the degree to which foreign capital inflows contribute to value added in the sector. This issue is of great importance for several related reasons. First, the agricultural sector, especially in developing and poor countries, draw their funding largely from the public sector. Second, even prior to the COVID-19 pandemic, such funding was often insufficient (see, for example, Borgomeo and Santos [9]) and the negative effects of the pandemic on public sector budgets are likely to exacerbate the problem. The fall in global capital flows, induced by the COVID-19 pandemic, is likely to compound the funding problems in the agricultural sector as well [38]. It is estimated that the annual financing gap for agricultural, small and medium enterprises (SMEs) in sub-Saharan Africa stands at USD 65 billion and in East Africa where 65% of the population works in the agricultural sector and accounts for twenty-five percent of GDP, and only 5% of commercial bank lending goes to the sector [39]. Globally, the amount of investments required to meet the sustainable development goals on food security is reported at about US$300billion per year [25]. Private and/or foreign investments are expected to contribute a significant share of this number, and understanding the extent of the contributions of these private investments to the food security is necessary for policies to reduce obstacles to private financing in the sector. Typical obstacles to investment in the sector include high transaction costs of access for populations in remote areas, fragmentation in agricultural value chains, which reduces investment demand, and lack of expertise at the level of financial institutions related to agricultural loan portfolios as well as a lack of appropriate instruments for managing risks in such a portfolio [56]. Third, the funding problems in the primary sector in developing economies are likely to be exacerbated by the current geopolitical conflict in Eastern Europe. The war in Ukraine and sanctions on Russia are having substantial negative food security impacts on countries in the Middle East, Africa, and elsewhere that rely on wheat imports from Ukraine and Russia [7, 41, 42], and food prices in global markets risking a 22% increase this year alone [16]. Both the COVID-19 pandemic and uncertainties associated with the current geopolitical events have implications for global supply chains, which themselves are closely linked to capital flows. For example, the conflict between Russia and Ukraine has disrupted wheat and fertilizer supplies in the world market, with Africa seen as the region most at risk (see [31]). Similarly, trade flows are expected to fall, with the World Trade Organization [55] projecting merchandise trade volume growth of 3% in 2022 compared to an earlier estimate of 4.7%, and approximately twenty percent of global air cargo is affected by airspace bans between Russia and other countries [22]. These have implications for trade, supply chains and foreign direct investment, especially in the agricultural sector due to the interdependencies between them. According to findings in Punthakey [44], FDI plays a crucial role in driving participation in agro-food global value chains. Similarly, Amendolagine et al. [3] find that FDI accounts for an intense global value chain participation and upstream specialization in Vietnam and sub-Saharan Africa. Furthermore, Alam and Bagchi [2] find that supply chain capability of a country is an important driver of FDI flows and that this effect varies by size of the host’s economy. This supports current projections that ongoing disruptions in supply chains will have negative effects on FDI flows and growth. This will reduce the ability of developing countries to achieve their sustainable development goals across various areas, for example gender inequality (see Fernandes and Kee [18] on the link between FDI, supply chain linkages and women empowerment). Furthermore, the general trend of trade disputes and restrictions will negatively affect FDI, even if the effects will be asymmetric [28].

Much of the current literature on FDI and agriculture focuses on food security or other development indicator; some of it consider disaggregated FDI and others use total FDI. A common weakness of this literature is the disregard for problems of endogeneity. To examine the role of FDI flows to agriculture, forestry and fishing, we apply a panel vector autoregression (or panel VAR) model that also allows us to address the potential issue of endogeneity. Specifically we consider FDI flows into this sector, partly motivated by the fact that the effect of FDI flows differs across sectors and industries (see “Literature” section). Our results show that there is a bidirectional causality between FDI in agriculture, forestry and value added by this sector. This bidirectional causality is present in most variables of our model. These bidirectional relationships reflect a cyclical effect between FDI and value added in the agriculture, forestry and fishing. These results imply that reducing these obstacles can have considerable benefits in the short and long term. The rest of the paper is organized as follows: We present a review of the literature in “Literature” section and discuss methods and data in “Methods and data” section. In “Results and discussion” section, we present and discuss the results and conclude in “Conclusions and discussions” section.

## Literature

The literature on the effects of FDI on the agricultural sector or food security remains scant, despite the importance of this sector as well as evidence on the significant rise in global capital flows into and out of various sectors of the world’s economies. Our work draws from to three, albeit limited strands of literature on this topic, all built on two theoretical foundations: the modernization thesis and dependency theory; both tied to the discourse on globalization. The modernization thesis holds that foreign capital inflows produce growth that benefits both the host country and the source country. On the other hand, the dependency theory posits that foreign capital inflows increase income inequality and reduce food security (see Mihalache-O'Keef and Li [36] for a summary of the supporting literature). The three strands of literature that motivate our analysis include the relationship between FDI and food security, FDI and food production, FDI and food security (or poverty, as well as other development indicators). The latter exploits the requirement that achieving the Sustainable Development Goal of zero hunger recognizes the paramount importance of investments in agriculture. Recognizing the links between various sustainable development goals, Dhahri and Omri [13] uses foreign direct investments and other forms of foreign assistance (such as social infrastructure aid, investment aid, agriculture-forestry-fishing aid and non-investment aid) reduce poverty and improve food security through their effects on the agricultural sector. They find that FDI has positive impacts on agricultural production, which in turn reduces food insecurity and poverty. In their three-step approach, no causal link running from agriculture to FDI is established.

The findings on the relationship between FDI and food security remain not just limited but mixed as well, with the former considered as overall FDI flows or only at the sectoral level. Authors such as Firebaugh and Beck [19], Jenkins and Scanlan [30] find positive effects of foreign capital on food security, while others Wimberley and Bello [54], Wimberley [53] find a negative relationship between food security and FDI. The results for the literature focusing on sectoral FDI are also mixed. Mihalache-O'Keef and Li [36] were the first to consider disaggregated FDI and its effects on food security. Specifically, they study three types of FDI: primary sector FDI, manufacturing FDI and service-sector FDI. Using data for 56 developing and transition economies, they find that manufacturing sector FDI improves food security. They find, however, that primary sector FDI reduces food security, while the effects of service-sector FDI on food security ranges from negative to ambiguous. Their results on the effect of primary sector FDI, though counterintuitive, is to be expected since much of the FDI into this sector tends to be focused on extractive activity rather than agricultural investment and growth. In our paper, we focus specifically on FDI in the agriculture, forestry and fishing and consider value added rather than food security as our outcome variable of interest. These results are partially in line with those obtained by Ben Slimane et al. [8] who consider FDI in agriculture, secondary and tertiary sectors in fifty-five countries during the period 1995–2009. They find that agricultural sector FDI improves food security, while FDI in the secondary and tertiary sectors reduces food security. While Ben Slimane et al. [8] examine agricultural FDI and food security, our paper considers the broader sector of agriculture to include forestry and fishing, and our outcome variable is value added in the sector rather than food security. Santangelo [45] studies the effect of FDI on food security by focusing on the most recent controversial aspects of foreign capital flows from rich to poor but resource-rich countries, specifically targeting the choicest of land (commonly referred to as ‘land grabbing’) in these countries. The study, using a sample of 65 developing economies for the period 2000–2011, finds that FDI in land originating from developed economies positively affects food security but that the opposite is true for FDI in land flowing from developing economies.

Empirical evidence on the relationship between FDI and poverty can depend on sample selection, methods and the measure of poverty used. Magombeyi et al. [34] uses a time-series model to examine the relationship between FDI and poverty reduction in Botswana from 1980 to 2014. However, their results were found to be sensitive to measures of poverty, with FDI having a short-run positive impact on poverty reduction but a negative long-run impact in the long run when life expectancy is used as a poverty reduction measure. Neither short-run nor long-run statistically significant effects of FDI are observed for mortality rate as a poverty reduction proxy. However, short-run negative impact of FDI on poverty reduction is confirmed when household consumption expenditure is used as a poverty reduction proxy, while an insignificant relationship is reported for the long run. Their results are mostly in line with Gohou and Soumaré [21], who investigate the same relationship for the period 1990–2007 for African countries and find strong positive relationship between FDI and welfare measures, specifically the human development index published by the United Nations Development Program (UNDP). A related topic is the relationship between foreign aid and food security (see Dhahri and Omri [13] for a brief outline of this literature), whose empirical evidence remains mixed.

Our work is most related to the strand of literature focusing on the relationship between FDI and agriculture. Findings in the existing literature, while mixed, generally support the view that FDI has positive effects on agriculture. Furtan and Holzman [20] examine the effect of FDI in the Canadian agriculture and food industry and find positive effects of FDI on the level of agriculture and food trade. Edeh et al. [15] use quarterly data for the period 1981–2017 to study the impact of foreign direct investment on the agriculture sector in Nigeria. They find that FDI has a positive and significant impact on the output of the agricultural sector and that this impact is stronger in the short run than in the long run. In a study of how sectoral FDI inflows affect growth of respective sectors in India, Jana et al. [29] find that FDI inflows do not contribute to output growth in the agricultural sector. Interestingly, they find a reverse causality wherein agricultural output attracts more FDI into the sector. In a study of the effect of FDI on sectoral growth, Opoku et al. [40] find that the pass-through impact of FDI is significant for the agricultural and service sectors. Furthermore, Tondl and Fornero [49] investigate the productivity effects of FDI in different sectors in Latin America, including agriculture and find agriculture to be one of the sectors where direct productivity effects are highest. The effect of FDI on agriculture manifests via various channels. Walkenhorst [52] investigates these channels by examining the impact of foreign investment in the sugarbeet-processing industry on the wider agro-food sector, using data on Central European transition economies. Empirical findings of the study indicate that foreign direct investment brings not only much needed capital to the region but also managerial and technological skills which are in similarly short supply. Technical support in the form of training programmes, pilot demonstration projects and innovative contract designs is found to help foreign affiliates secure sufficient high-quality raw material supplies, while inducing sector-wide improvements in agricultural productivity and agri-business practices.’

Though limited, a similar strand of the literature examines the relationship between foreign aid and agricultural production or output. McArthur and Sachs [35] illustrate via simulations of a general equilibrium model that official development assistance (ODA) for agriculture is capable of generating an expansion in the primary tradable sector and positive permanent productivity. Dhahri and Omri [12] examine the effect of FDI and various forms of aid on agricultural output using data for 50 developing countries over the 1995–2015 period. They find that FDI alone (excluding foreign aid) has a positive and statistically significant effect on agricultural production. They further find that all four forms of foreign aid considered (social-infrastructure aid, investment aid, non-investment aid, and agriculture–forestry–fishing aid) also have positive and significant effects on agricultural production. However, only social-infrastructure aid and agriculture–forestry–fishing aid maintain their effects on agricultural output when all forms of aid and FDI are included in the same estimation. Kherallah et al. [32] apply a simultaneous equation model to data on 56 developing countries for the period 1974–1990 to study the relationship between foreign aid and agricultural growth, finding a positive and statistically significant link between foreign aid and agricultural growth, where a 1% increase in foreign aid is associated with a 0.75% growth in agriculture. Norton et al. [37] use data on a sample of 98 less developed countries from 1970 to 1985 to study the impact that foreign aid has on agriculture productivity. Their results differ across various geopolitical groupings or regions and countries, with aid enhancing agricultural productivity in Asia and sub-Saharan Africa, while Latin America and the Middle East experience a negative impact of foreign aid on agricultural productivity. They also find that aid appears to be less effective in countries with large fiscal deficits and high external debt. Instead of using general foreign aid as in Norton et al. [37] and Kherallah et al. [32], Barkat and Alsamara [6] use data for 29 African countries over the period of 1975–2013 to examine the impact of foreign agricultural aid and foreign aid on agricultural output. Their results show a small and positive impact of foreign agricultural aid and total foreign aid on agricultural output for low- and middle-income countries. Their further assessment of the data reveals evidence of a bidirectional causal relationship between agricultural aid and agricultural output. Similarly, Dewbre et al. [11] use data on 87 developing countries for the period 1985 to 2004 to study the relationship between agricultural aid flows and agricultural growth. Contrary to other literature, they found that agricultural aid flows negatively affect agriculture growth. Rather than aid flows and agricultural growth, our paper focuses FDI and value added in a broader agricultural sector that includes forestry and fishing.

## Methods and data

Given the numerous strands of literature on the role of FDI in the economy, a wide variety of methods has been used in the literature. Time-series methods are commonly used to examine the relationship between FDI and agricultural growth. The choice of a particular method aims at addressing some of the intractable problems associated with the characteristics of the data. In particular, the problem of endogeneity remains underappreciated in the current literature. In this paper, we use the panel vector autoregression model, which treats our variables of interest in the model as endogenous. The model, first introduced by Holtz-Eakin et al. [26], is widely used in analyzing the spillover effects of macroeconomic and financial shocks. We use the model to account for possible endogeneity, supported by existing evidence in the literature. Specifically, Jana et al. [29] find a reverse causality wherein agricultural output is found to be attracting more FDI into the sector. This is in line with other sectors as well; for example, Srikanth and Kishore [46] established bidirectional causality from FDI and industrial production of India measured by Index of Industrial Production. Furthermore, Awunyo-Vitor and Sackey [5] find a statistically significant and positive relationship between economic growth and foreign direct investment flows to the agricultural sector, implying that FDI flows into the sector simultaneously affect two variables of our model. Applying the panel vector autoregression model helps us bypass the difficult problem of choosing valid instruments in alternative estimations. A multivariate panel vector autoregression of order 1 with country-specific fixed effects can be expressed as:

1 $${Y}_{it}={\beta }_{0}+{\beta }_{1}{Y}_{i, t-1}+{\beta }_{2}{X}_{i,t}+{U}_{i,t}+{\varepsilon }_{i,t}$$

where Y_it_ represents an n-variable vector and X_i,t_ is a vector of exogenous variables. ${U}_{i,t}$ and ${\varepsilon }_{i,t}$ are dependent-variable-specific fixed effects and idiosyncratic errors, respectively. β_0_ and β_1_ are (*1* × *k*) and (*k* × *k*) vectors of parameters to be estimated, where *k* is the number of parameter estimates. We use a 4-variable vector consisting of value added, FDI, official development, and gross fixed capital formation in agriculture, forestry and fishing. We use the share of value added in gross domestic product from agriculture, forestry and fishing as an exogenous variable in the model. The model allows us to examine the dynamic relationship between FDI in agriculture, forestry and fishing and the performance of this sector (using value added as a proxy for performance). It also estimates fixed effects by augmenting a standard vector autoregression with a cross-country dimension. This is done via the system generalized method of moments (GMM) estimation, which uses lagged values as instruments to address endogeneity and provide unbiased estimates. The lag length we use is based on the model selection criteria laid out by Abrigo and Love [1], who derive criteria based on the guidelines of model selection for GMM estimation by comparing the J-statistics obtained from testing the over-identifying restrictions on the number of parameters of interest. We follow Abrigo and Love [1] by using the Bayesian information criteria (BIC), Hann–Quinn information criteria (QIC), and Akaike information criteria (AIC). These metrics capture the trade-off between over-specification and precision. The goal is to choose the lag order that minimizes these metrics, which for our model is the first-order lag.

### Data

All our data come from the Food and Agricultural Organization database.^1^ We collect data on sixteen developing countries over the period 2001–2020.^2^ Our FDI variable is represented by foreign direct investment inflows in agriculture, forestry and fishing. According to the International Monetary Fund [27], FDI refers to ‘an investment involving a long-term relationship and reflecting a lasting interest and control by a resident entity in one economy (foreign direct investor or parent enterprise) in an enterprise resident in an economy other than that of the foreign direct investor (FDI enterprise or affiliate enterprise or foreign affiliate.’

We consider the annual growth (in US$) rate of this variable, rather than its levels. Agriculture, forestry and fishing is represented by the value added (VA) in these three subsectors sector (measured in US$ 2015 prices in millions).

We include official development assistance (DFA) or flows in agriculture, forestry and fishing. According to FAO [17], these are ‘flows to countries and territories, which are provided by official agencies, including state and local governments, or by their executive agencies, and each transaction of which: is administered with the promotion of the economic development and welfare of developing countries as its main objective.’ The data on this variable are measured in millions US$, 2019 prices. This variable helps reduce the likelihood of misidentifying any observed effects of FDI on agriculture, fishing and forestry. To account for investments and attractiveness of agriculture, forestry and fishing, we include fixed capital formation (GFC) in this sector. According to FAO [17], ‘capital in the agriculture sector includes the machinery, equipment and tools as well as the farm buildings, and is essential in the production of all agricultural outputs. The gross fixed capital formation is an indication of the amounts that are reinvested in new fixed assets that are part of capital.’ To control for the size of the agriculture, forestry and fishing as a proxy for the absorptive capacity for the growth is share of value added in GDP (AFFGDP), expressed in percentage. The summary statistics of these variables are presented in Table 1.

**Table 1:** Summary statistics

Variable	Obs	Mean	Std. dev	Min	Max
VA	320	21,538.16	25,799.46	250.3882	139,998.7
FDI	320	− 365.232	9064.165	− 161,700	6500
DFA	320	1.460547	0.7058823	− 2.30698	2.713789
AFFGDP	320	10.53824	5.69123	2.784589	27.47565
GFC	320	5.044757	14.05692	− 44.1989	133.8619

Table 1 shows large variances in FDI flows and value added in agriculture, forestry and fishing. We then transform our variables that expressed at levels into logarithmic form and de-trend each variable in the model. We then implement a Harris–Tzavalis unit root test, and the presence of stationarity is validated for all four variables. The results of the unit-root tests are presented in Table 2, showing that the null hypothesis of the presence of a unit root is rejected. Therefore, all the variables used in the panel-VAR are stationary. The null hypothesis in the Harris–Tzavalis unit root test is that panels contain unit roots, and the alternative is that panels are stationary.

**Table 2:** Harris–Tzavalis unit root test

Variable	Number of panels	Number of periods	Statistic: rho	*Z* value	*P* value
VA	16	20	0.7353	− 3.295	0.0005
FDI	16	20	− 0.058	− 24.7455	0.0000
DFA	16	20	0.2838	− 15.5027	0.0000
AFFGDP	16	20	0.6929	− 4.4405	0.0000
GFC	16	20	− 0.1192	− 26.402	0.0000

## Results and discussion

The results we report use a system GMM-style estimation of the panel-VAR. It fits homogeneous panel-VAR model by fitting a multivariate panel regression of each dependent variable on lags of itself and on lags of all other dependent variables using generalized method of moments [1]. We follow the standard analytical process for a panel-VAR models. Specifically, Granger causality is first tested, stability of the model is established, impulse response functions and variance decomposition measures are obtained. First, the results of the Granger causality test are presented in Table 3. In this test, we are interested in whether past values of one variable (say FDI in our model) can be used to predict values of another variable (say VA in our model), conditional on the past values of the latter – that is, whether FDI Granger-causes VA [23]. Each row in Table 3 represents an equation. For example, for FDI, the equation shows the test on whether the coefficients on the lag of each of VA, DFA, GFC in the FDI equation are statistically different from zero. The label ‘ALL’ is with respect to the coefficients of all lags of all endogenous variables other than those of the dependent variable being jointly zero. The null hypothesis that VA, DFA, GFC individually and collectively does not Granger-cause FDI is rejected at levels between 1 and 5%. Similarly, the null hypothesis that FDI, DFA, GFC individually and collectively does not Granger-cause VA is rejected at levels between 1 and 5%. According to the results of the Granger causality test in Table 3, there is a bidirectional causality between FDI in agriculture, forestry and value added by this sector. Except for DFA and FDI, DFAA and GFC, VA and GFC, the bidirectional causality is present in all other variables of our model. These bidirectional relationships reflect a cyclical effect between FDI and value added in the agriculture, forestry and fishing and the rest of the variables in the model.

**Table 3:** Panel VAR-Granger causality Wald test

Ho: Excluded variable does not Granger-cause equation variable			
Ha: Excluded variable Granger-causes equation variable			
Equation\Excluded	Chi2	d.f	Prob > chi2
*FDI*			
VA	14.322	1	0.0000
DFA	12.284	1	0.0000
GFC	4.856	1	0.028
ALL	22.512	3	0.0000
*VA*			
FDI	70.468	1	0.0000
DFA	8.47	1	0.004
GFC	4.377	1	0.036
ALL	93.032	3	0.0000
*DFA*			
FDI	0.858	1	0.354
VA	13.885	1	0.0000
GFC	0.249	1	0.618
ALL	14.846	3	0.002
*GFC*			
FDI	9.409	1	0.002
VA	1.721	1	0.19
DFA	7.735	1	0.005
ALL	15.099	3	0.002

We can examine the dynamic relationships between the variables of our model with the help of impulse response functions and variance decomposition measures. This requires that that the panel-VAR be invertible and has an infinite-order vector moving average representation [1]. We can establish this by examining the stability condition, which grants validity to any dynamic inferences we make about our model. Specifically, after fitting panel VAR, the moduli of the companion matrix based on the estimated parameters can be estimated. This is shown in Table 4. We require that all moduli be smaller than one for the model to be stable.

**Table 4:** Eigenvalue stability condition

Eigenvalue		
Real	Imaginary	Modulus
9,572,009	0	0.9572009
− 0.3501007	0	0.3501007
0.0840641	0	0.0840641
0.0220289	0	0.0220289

**Fig. 1:**
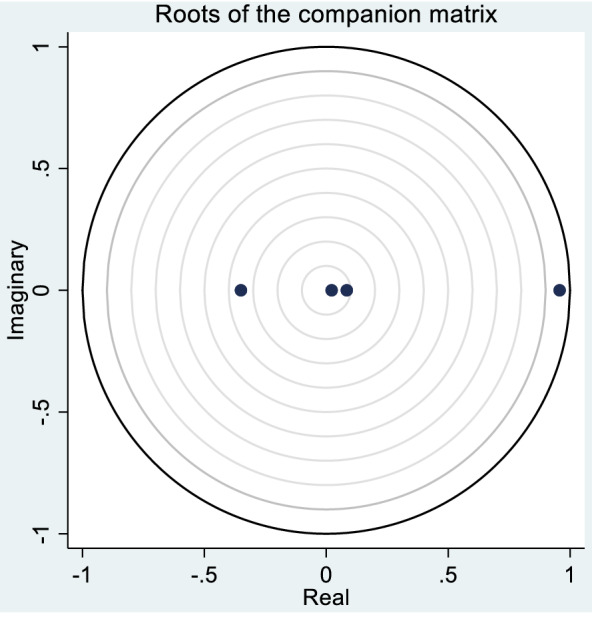
Stability condition

We conclude that our model is stable because all the moduli are smaller than one. The stability condition can be graphically illustrated, as given in Fig. 1. In Fig. 1, model stability requires that the roots of the companion matrix all fall inside the unit circle. This stability allows us to be able to compute the impulse response functions as well as variance decomposition (see Table 6 in Appendix).

**Fig. 2:**
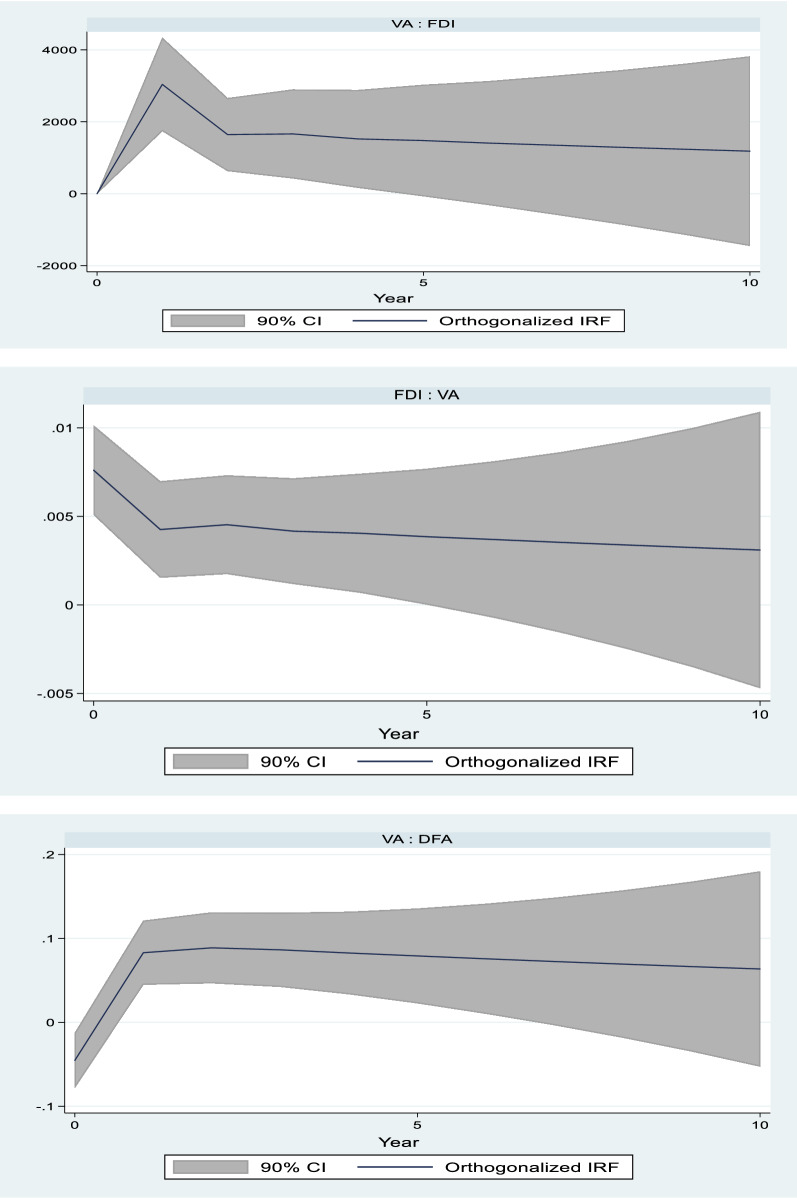
Impulse response functions

There is no empirical test for the ordering in panel-VAR estimation. Instead, we simply use the ordering of variables specified in panel VAR estimation procedure. The impulse response (IRF) functions for our estimation are shown in Fig. 2, and the GMM estimation is shown in Table 7 in appendix. In the results, we focus only on the most two important variables of interest in our analysis: FDI and value added in agriculture, forestry and fishing. In addition, the impulse response functions (Fig. 2) present the main results to be interpreted. Figure shows the causal effects between FDI and value added in agriculture, forestry and fishing. According to the results, the effect of FDI on value added in agriculture, forestry and fishing is not immediate but rises and remains a positive for up to five years in our model. This implies FDI has a medium- to long-term positive impact on value added in agriculture, forestry and fishing.

Similarly, value added in agriculture, forestry and fishing serves as a pull factor to FDI, and this effect persists in the medium to long term. The relationship between value added and development assistance shows positive and self-reinforcing effects. Another variable with strong explanatory power in the model is the development assistance in agriculture, forestry and fishing. According to Fig. 2, only after value added has achieved a certainly level of performance does development assistance begins to have a statistically significant impact on agriculture, forestry and fishing.

## Conclusions and discussions

The COVID-19 pandemic and current geopolitical events have created high levels of uncertainty in food chains across the globe, and this issue will increasingly pull the agricultural sector into focus in policy circles for governments and international bodies. Among the factors that will likely be focused on is the role of investment and technology in the agricultural sector. FDI is a crucial determinant of agricultural production via technology transfer and skills that benefit host country’s farmers. Empirical analyses on this topic, especially in the context of the global pandemic and geopolitical conflict, remain limited. The goal of this paper is to contribute to the discourse. Specifically, we aim to examine the role of FDI and performance in agriculture, forestry and fishing. To achieve this, we apply a panel VAR model to data on sixteen developing countries over a twenty-year period. This model is particularly suitable because of its ability to deal with the problem of endogeneity. Our results show that FDI have medium- to long-term positive effects on value added in agriculture, forestry and fishing. This suggests that developing countries would benefit from eliminating policies that and other regulations that increase transaction costs for foreign investments. This includes improving institutional mechanisms that deter foreign investments—Amendolagine et al. [4] as well as Hasan et al. [24] lend support to the argument that good institutions boost foreign direct investments and enhancing the efficient management of public sector budgets. Public policy can also work to create a conducive environment that facilitate corporate treasury management to increase corporate effectiveness and productivity, which in turn would draw more investments (see Polak et al. [43] for analysis on treasury management). Such improvements can help developing countries attract FDI in sector in which they currently underperform, for example in advanced business services, which require considerably high levels of skilled labor [33].

## Data Availability

The dataset used in the study is available in the Food and Agricultural Organization database: https://www.fao.org/faostat/en/#data/FDI. The data for our analysis is available from the corresponding author on reasonable request.

## Appendix

See Tables 5, 6 and 7.

**Table 5:** Countries in the sample

Bangladesh
Chile
Colombia
Costa Rica
Ecuador
Guatemala
Honduras
Indonesia
Malaysia
Mauritius
Mexico
Mozambique
Paraguay
Thailand
Turkey
Viet Nam

**Table 6:** Variance decomposition

Response variable and forecast horizon	Impulse variable			
	FDI	VA	DFA	GFC
*FDI*				
0	0	0	0	0
1	1	0	0	0
2	0.8055624	0.0546782	0.1305487	0.0092107
3	0.7869866	0.0755716	0.128301	0.0091408
4	0.7688392	0.0933242	0.1284195	0.0094171
5	0.7548875	0.1085172	0.1272704	0.0093249
6	0.7420815	0.1220162	0.1265758	0.0093265
7	0.730903	0.1339534	0.1258464	0.0092972
8	0.7209082	0.1445763	0.1252346	0.0092809
9	0.7120129	0.1540474	0.1246768	0.009263
10	0.7040535	0.1625162	0.124182	0.0092482
*VA*				
0	0	0	0	0
1	0.0716618	0.9283382	0	0
2	0.0429272	0.8907034	0.0580759	0.0082936
3	0.037063	0.8926349	0.0627992	0.0075029
4	0.0336507	0.890612	0.0679352	0.007802
5	0.0318229	0.8902867	0.0701147	0.0077756
6	0.0305822	0.8898315	0.0717713	0.0078151
7	0.0297245	0.8895843	0.0728654	0.0078258
8	0.029087	0.8893798	0.0736944	0.0078388
9	0.0285999	0.88923	0.0743229	0.0078472
10	0.0282158	0.8891097	0.0748201	0.0078543
*DFA*				
0	0	0	0	0
1	0.00896	0.0058009	0.9852391	0
2	0.0104409	0.0614479	0.9271096	0.0010016
3	0.0114125	0.1166953	0.8705162	0.001376
4	0.0120703	0.1620835	0.8240482	0.0017981
5	0.0126308	0.1992441	0.7860222	0.0021028
6	0.0130877	0.2301082	0.7544356	0.0023685
7	0.0134744	0.256052	0.7278859	0.0025878
8	0.0138019	0.2780876	0.7053351	0.0027753
9	0.0140829	0.2969691	0.6860125	0.0029356
10	0.0143254	0.31327	0.6693305	0.0030741
*GFC*				
0	0	0	0	0
1	0.0366768	0.0485134	0.0656898	0.84912
2	0.0357273	0.0397243	0.2294772	0.6950713
3	0.035864	0.0388171	0.2385513	0.6867676
4	0.0358491	0.0387668	0.2398525	0.6855315
5	0.0358563	0.0387576	0.2399733	0.6854128
6	0.035854	0.0387728	0.2399969	0.6853763
7	0.0358545	0.0387832	0.2399942	0.685368
8	0.0358541	0.0387944	0.2399934	0.6853581
9	0.0358541	0.0388042	0.2399912	0.6853504
10	0.0358539	0.0388134	0.2399897	0.6853431

**Table 7:** Panel vector autoregression GMM estimation

	Coeff	Stad Err	Z	P >|z|
*FDI*				
FDI_t-1_	− 0.0070339	0.014192	− 0.5	0.62
VA_t-1_	94903.57	25077.41	3.78	0.0000
DFA_t-1_	− 11184.71	3191.266	− 3.5	0.0000
GFC_t-1_	− 71.00288	32.21997	− 2.2	0.028
AFFGDP_t_	− 745.8474	410.6999	− 1.82	0.069
*VA*				
FDI_t-1_	− 2.11E−07	2.52E−08	− 8.39	0.0000
VA_t-1_	1.058401	0.0999634	10.59	0.0000
DFA_t-1_	− 0.0269734	0.009268	− 2.91	0.004
GFC_t-1_	− 0.0002521	0.0001205	− 2.09	0.036
AFFGDP_t_	− 0.0117889	0.002094	− 5.63	0.0000
*DFA*				
FDI_t-1_	− 6.00E−07	6.48E−07	− 0.93	0.354
VA_t-1_	3.123305	0.8381881	3.73	0.0000
DFA_t-1_	0.0079548	0.1044854	0.08	0.939
GFC_t-1_	− 0.0007275	0.0014581	− 0.5	0.618
AFFGDP_t_	0.1124806	0.021866	5.14	0.0000
*GFC*				
FDI_t-1_	− 0.0000634	0.0000207	− 3.07	0.002
VA_t-1_	72.98603	55.62858	1.31	0.19
DFA_t-1_	− 20.0784	7.219461	− 2.78	0.005
GFC_t-1_	− 0.3461282	0.0791308	− 4.37	0.0000
AFFGDP_t_	4.242586	1.141165	3.72	0.0000

## Abbreviations

FDI: Foreign direct investment
VAR: Vector autoregression
GDP: Gross domestic product
UNCTAD: United Nations conference on trade and development
FAO: Food and agricultural organization
OECD: Organization for Economic Cooperation and Development
UNDP: United Nations Development Program
ODA: Official development assistance
GMM: Generalized method of moments
WTO: World Trade Organization

## Footnotes

1 https://www.fao.org/faostat/en/#data.

2 See Table 5 in the Appendix for a list of the countries included in our sample.

## References

[1] Abrigo M, Love I (2016). Estimation of panel vector autoregression in Stata. Stata J.

[2] Alam A, Bagchi PK (2011). Supply chain capability as a determinant of FDI. Multinatl Bus Rev.

[3] Amendolagine V, Presbitero AF, Rabellotti R, Sanfilippo M (2019). Local sourcing in developing countries: the role of foreign direct investments and global value chains. World Dev.

[4] Amendolagine V, Boly A, Coniglio N, Prota F, Seric A (2013). FDI and local linkages in developing countries: evidence from sub-Saharan countries. World Dev.

[5] Awunyo-Vitor D, Sackey RA (2018). Agricultural sector foreign direct investment and economic growth in Ghana. J Innov Entrep.

[6] Barkat K, Alsamara M (2019). The impact of foreign agricultural aid and total foreign aid on agricultural output in African countries: new evidence from panel data analysis. S Afr J Econ.

[7] Behnassi M, El Haiba M (2022). Implications of the Russia–Ukraine war for global food security. Nat Hum Behav.

[8] Ben Slimane M, Huchet-Bourdon M, Zitouna H (2016). The role of sectoral FDI in promoting agricultural production and improving food security. Int Econ.

[9] Borgomeo E, Santos N (2019) Towards a new generation of policies and investments in agricultural water in the Arab region: fertile ground for innovation. FAO, Rome, International Water Management Institute (IWMI), Colombo. p 124. https://www.fao.org/documents/card/en/c/ca4445en/

[10] Castañeda A, Doan D, Newhouse D, Nguyen MC, Uematsu H, Azevedo JP (2016) Who are the poor in the developing world? Policy research working papers. October 2016. 10.1596/1813-9450-7844

[11] Dewbre J, Thompson W, Dewbre J (2007). Consistency or conflict in OECD agricultural trade and aid policies. Am J Agr Econ.

[12] Dhahri S, Omri A (2020). Does foreign capital really matter for the host country agricultural production? Evidence from developing countries. Rev World Econ.

[13] Dhahri S, Omri A (2020). Foreign capital towards SDGs 1 & 2—ending poverty and hunger: the role of agricultural production. Struct Chang Econ Dyn.

[14] Dumas P, Hanson C, Ranganathan J, Searchinger T, Waite R (2019) Creating a sustainable food future. World Resources Institute, Washington, ISBN 9781569739631. https://research.wri.org/wrr-food

[15] Edeh CE, Eze CG, Ugwuanyi SO (2020). Impact of foreign direct investment on the agricultural sector in Nigeria (1981–2017). Afr Dev Rev.

[16] FAO (2022). The importance of Ukraine and the Russian Federation for global agricultural markets and the risks associated with the current conflict.

[17] FAO (2021). World Food and Agriculture - Statistical Yearbook 2021. Rome.

[18] Fernandes AM, Kee HL (2021). Women empowerment, supply chain linkages and FDI: evidence from Bangladesh. Transnational Corporations.

[19] Firebaugh G, Beck FD (1994). Does economic growth benefit the masses? Growth, dependence, and welfare in the third world. Am Sociol Rev.

[20] Furtan WH, Holzman JJ (2004) The effect of FDI on agriculture and food trade: an empirical analysis. Agriculture and Rural Working Paper Series. Working Paper No. 68

[21] Gohou G, Soumaré I (2012). Does foreign direct investment reduce poverty in Africa and are there regional differences?. World Dev.

[22] Guenette JD, Kenworthy PG, Wheeler CM (2022). Implications of the War in Ukraine for the Global Economy. EFI Policy Note 3. World Bank, Washington. https://openknowledge.worldbank.org/handle/10986/37372

[23] Granger CWJ (1969). Investigating causal relations by econometric models and cross-spectral methods. Econometrica.

[24] Hasan M, Rahman MN, Iqbal BA (2017). Corruption and FDI inflows: evidence from India and China. Mediterr J Soc Sci.

[25] Havemann T, Negra C, Werneck F (2020). Blended finance for agriculture: exploring the constraints and possibilities of combining financial instruments for sustainable transitions. Agric Hum Values.

[26] Holtz-Eakin D, Newey W, Rosen H (1988). Estimating vector autoregressions with panel data. Econometrica.

[27] IMF (International Monetary Fund) (1993) Balance of payments manual 5th edition. International Monetary Fund, Washington

[28] Iqbal BA, Rahman N, Elimimian J (2019). The future of global trade in the presence of the Sino-US trade war. Econom Polit Stud.

[29] Jana SS, Sahu TN, Pandey KD (2019). Foreign direct investment and economic growth in india: a sector-specific analysis. Asia-Pacific J Manag Res Innov.

[30] Jenkins JC, Scanlan SJ (2001). Food security in less developed countries, 1970 to 1990. Am Sociol Rev.

[31] Kennes DJ, Taylor S, Fonseca B (2022) The Russia–Ukraine war’s impact on global fertilizer markets. RaboResearch Food and Agribusiness, Rabobank. https://research.rabobank.com/far/en/sectors/farm-inputs/the-russia-ukraine-war-impact-on-global-fertilizer-markets.html

[32] Kherallah MW, Beghin JC, Peterson EWF, Ruppel FJ (1994). Impacts of official development assistance on agricultural growth, savings and agricultural imports. Agric Econ.

[33] Klimek A (2020). Determinants of foreign direct investment in advanced business services. Acta Oeconomica.

[34] Magombeyi MT, Odhiambo NM, Watson D (2018). FDI inflows and poverty reduction in Botswana: an empirical investigation. Cogent Econ Finance.

[35] McArthur JW, Sachs JD (2019). Agriculture, aid, and economic growth in Africa. World Bank Econ Rev.

[36] Mihalache-O’Keef A, Li Q (2011). Modernization vs. dependency revisited: effects of foreign direct investment on food security in less developed countries. Int Stud Q.

[37] Norton GW, Ortiz J, Pardey PG (1992). The impact of foreign assistance on agricultural growth. Econ Dev Cult Change.

[38] Nyiwul L (2021). COVID-19 regulatory responses and FDI in the United States: trends and implications for capital flows. Transnatl Corp Rev.

[39] OECD/UNCDF (2020). Blended finance in the least developed countries 2020: supporting a resilient COVID-19 recovery.

[40] Opoku EEO, Ibrahim M, Sare YA (2019). Foreign direct investment, sectoral effects and economic growth in Africa. Int Econ J.

[41] Osendarp S, Verburg G, Bhutta Z, Black RE, de Pee S, Fabrizio C, Headey D, Heidkamp R, Laborde D, Ruel MT (2022). Act now before Ukraine war plunges millions into malnutrition. Nature.

[42] Parker C (2022) Five countries hit hard by the grain crisis in Ukraine, Washington Post. https://www.washingtonpost.com/world/2022/06/15/ukraine-war-russia-grain-food-crisis-world-hunger/

[43] Polak P, Masquelier F, Michalski G (2018). Towards treasury 4.0/The evolving role of corporate treasury management for 2020. Management.

[44] Punthakey J (2020) Foreign direct investment and trade in agro-food global value chains. OECD Food, Agriculture and Fisheries Papers, No. 142, OECD Publishing, Paris. 10.1787/993f0fdc-en

[45] Santangelo GD (2018). The impact of FDI in land in agriculture in developing countries on host country food security. J World Bus.

[46] Srikanth M, Kishore B (2012). FDI and its impact on Indian economy: an empirical investigation. Foreign Trade Rev.

[47] Stewart R, Langer L, Da Silva NR, Muchiri E, Zaranyika H, Erasmus Y, Randall N, Rafferty S, Korth M, Madinga N, de Wet T (2015). The effects of training, innovation and new technology on African smallholder farmers’ economic outcomes and food security: a systematic review. Campbell Syst Rev.

[48] Smith P, Bustamante M, Ahammad H, Clark H, Dong H, Elsiddig EA, Haberl H, Harper R, House J, Jafari M, Masera O, Mbow C, Ravindranath NH, Rice CW, Robledo Abad C, Romanovskaya A, Sperling F, Tubiello F, Edenhofer O, Pichs-Madruga R, Sokona Y, Farahani E, Kadner S, Seyboth K, Adler A, Baum I, Brunner S, Eickemeier P, Kriemann B, Savolainen J, Schlömer S, von Stechow C, Zwickel T, Minx JC (2014). Agriculture, forestry and other land use (AFOLU). Climate change 2014: mitigation of climate change. Contribution of working group III to the fifth assessment report of the intergovernmental panel on climate change.

[49] Tondl G, Fornero JA (2010) Sectoral productivity and spillover effects of FDI in Latin America (No. 53). FIW Working Paper

[50] Townsend R (2015). Ending poverty and hunger by 2030: an agenda for the global food system.

[51] UNCTAD (2018) The least developed countries report 2018. https://unctad.org/system/files/official-document/ldcr2018_en.pdf

[52] Walkenhorst P (2000). Foreign direct investment, technological spillovers and the agricultural transition in Central Europe. Post-Communist Econ.

[53] Wimberley DW (1991). Transnational corporate investment and food the third world: a cross-national analysis. Rural Sociol.

[54] Wimberley DW, Bello R (1992). Effects of foreign investment, exports, and economic growth on third world food consumption. Soc Forces.

[55] WTO (2022) Trade statistics and outlook. Russia–Ukraine conflict puts fragile global trade recovery at risk. Press Release Press/902. https://www.wto.org/english/news_e/pres22_e/pr902_e.htm

[56] World Bank (2020) Agriculture finance and agriculture insurance, Brief, October 8, 2020. https://www.worldbank.org/en/topic/financialsector/brief/agriculture-finance

